# Point of care testing to monitor INR control in patients with antiphospholipid syndrome

**DOI:** 10.1002/jha2.522

**Published:** 2022-07-09

**Authors:** Michael Masucci, Annabelle Li Kam Wa, Emilia Shingleton, Jonathan Martin, Zahra Mahir, Karen Breen

**Affiliations:** ^1^ GKT School of Medical Education King's College London London UK; ^2^ Haemostasis and Thrombosis Department Guy's and St Thomas' NHS Foundation Trust London UK

**Keywords:** Antiphospolipid Syndrome, antiphospholipid antibodies, international normalised ratio, thrombosis, warfarin

## Abstract

Patients with antiphospholipid syndrome (APS) typically require lifelong warfarin anticoagulation following a thrombotic event due to a significant risk of recurrent thrombosis. Point of care testing (POCT) to monitor INR is discouraged in patients with APS as interactions between antiphospholipid antibodies and thromboplastin used for INR testing may influence results. Review of INR testing in 36 APS patients showed 87.2% of paired POCT and venous INRs (*n* = 94) having acceptable variation (≤0.5 difference), and high correlation (*r* = 0.9) excluding INRs ≥4.8. Six‐month TTR was comparable for APS patients using POCT (57.1% ± 24.8%) to those using venous INR monitoring (59.2% ± 23.2%) (*p* = 0.66). These results support POCT management of APS but requires further study.

## 1 INTRODUCTION

Antiphospholipid syndrome (APS) is a systemic autoimmune disorder with thrombotic and/or obstetric complications in association with persistent antiphospholipid antibodies (APA) [1]. Clinical manifestations include both arterial and venous thrombosis as well as pregnancy related complications such as foetal loss, pre‐eclampsia and severe eclampsia [2].

Current guidelines recommend that patients with APS‐associated thrombosis have lifelong anticoagulation to prevent recurrence of thrombotic events [3]. This is typically with a vitamin K antagonist (VKA) such as warfarin. The therapeutic range at which VKAs offer protection from thrombosis varies with individual risk, but the current consensus is a target international normalised ratio (INR) of 2.0–3.0 for patients with APS [4]. This can be higher in those who experience thrombotic events whilst on treatment [4, 5].

Warfarin has an unpredictable anticoagulant effect and requires regular monitoring of the INR to maintain adequate dosing. This has typically been in phlebotomy clinics using venous sampling, however the use of point of care testing (POCT) has become more widely used in recent years. Use of POCT is discouraged in APS following concerns of a potential interaction between APA and POCT reagents, in particular lupus anticoagulant (LAC) with commercial thromboplastins [6]. Evidence suggests 10%–20% of patients positive for LAC may have falsely high INRs, which is more apparent using POCT as opposed to venepuncture [7]. This may falsely influence results and lead to subtherapeutic warfarin dosing.

The COVID‐19 pandemic has highlighted the many benefits of POCT to self‐monitor INRs. Minimising hospital visits reduces the strain on pressurised NHS services and prioritises patient safety by limiting spread of the virus. POCT is a key facilitator in the accelerated transition to telemedicine, particularly in haematology where the remote management of long‐term conditions such as APS is foreseeable in future practice.

## 2 METHODS

The aim of our study was to review the use of POCT in patients with APS attending an anticoagulant service in a tertiary referral centre.

We conducted a retrospective analysis of patient data using the DAWN anticoagulation database at Guy's and St Thomas’ NHS Foundation Trust (GSTT) dated between July 2018 to February 2021. Thirty‐six patients with APS (6 male and 30 female, age range 27–79 years old) using Roche CoaguChek XS (Roche Diagnostics, Basel, Switzerland) POCT devices were identified. Consecutive comparative venous and POCT INRs were performed prior to implementing POCT for INR testing. Patients were encouraged to attend on a 3–6 monthly basis for comparative venous and POCT INR testing.

Paired (same day) INR results from venous laboratory and POCT methods were assessed for variation. An acceptable variation was determined to be ≤0.5 INR units [8]. The 6‐month time in therapeutic range (TTR) in this APS POCT cohort was compared to 2 control cohorts: 72 patients with APS (25 male and 47 female, age range 44–82 years old) using venous‐only INR monitoring, and 30 non‐APS patients with similar thrombotic indications (17 male and 13 female, age range 20–91 years old) using POCT. Statistical analysis was performed using IBM SPSS Statistics Version 26.0.

## 3 RESULTS

**FIGURE 1:**
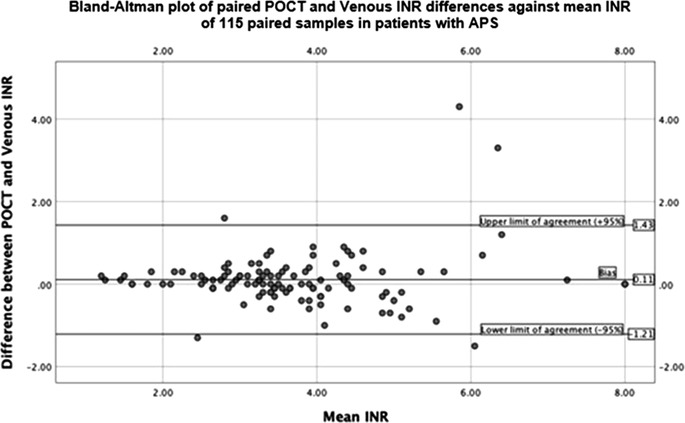
Bland‐Altman plot demonstrating the agreement between paired POCT and venous INRs. Limits of agreement are set at 95% confidence intervals

Thirty‐six patients with APS were found to have 115 paired venous and POCT INR samples, with a median of three paired samples per patient. 79.1% of these paired samples demonstrated an acceptable INR variation of ≤0.5. GSTT anticoagulation clinic policy is to recall patients reporting POCT INRs of ≥4.8 for a venous INR test since the accuracy of POCT results is not assured at higher readings [9]. 87.2% of 94 paired samples had an acceptable variation when POCT INRs of ≥4.8 were excluded. Correlation between measurements was assessed using Pearson product‐moment coefficients (*r*), showing a high correlation when excluding pairs with POCT INR ≥ 4.8 (*r* = 0.9), decreasing slightly for all 115 paired samples (*r* = 0.87). Bland‐Altman plots for all APS POCT paired samples (*n* = 115) showed high concordance between pairs (Figure 1), with 95.6% (*n* = 110) of pairs reliably between limits of agreement set at 95% confidence intervals. Additionally, we observed that as INR increased, concordance between methods decreased (Figure 1).

Mean 6‐month TTR for APS patients using POCT was 57.1% (±24.8), compared to both 59.2% (±23.2) in APS patients using venous testing only (*p* = 0.66) and 80.0% (±18.9) in non‐APS patients using POCT (*p* = 0.0002; two‐sample *T* test with unequal varices) (Table 1).

**TABLE 1:** Mean 6‐month TTR for APS patients using POCT compared to both APS patients using venous testing only and non‐APS patients using POCT, with *p* values determined by two‐sample *T*‐test with unequal varices

Cohort	Mean 6‐month TTR (% time)	Standard deviation	*p*
APS POCT (*n* = 36)	57.1	24.8	‐
APS venous‐only monitoring (*n* = 74)	59.2	23.2	0.66
Non‐APS POCT (*n* = 30)	80.0	18.9	0.0002

## 4 DISCUSSION

The interference of APA with INR testing reagents has raised concerns over the validity of POCT in APS management. This study assessed the reliability of CoaguChek XS POCT devices in monitoring patients with APS. Considering GSTT's recall policy for POCT results ≥4.8, 87.2% of paired samples showed an acceptable agreement when the INR was <4.8. This compares well to previously published figures of 84%–87% in reviews of CoaguChek XS against laboratory measurements in patients without APS [9, 10]. Similarly, Pearson correlation coefficient (*r*) of 0.9 was comparable to 0.966 achieved in the review by Kalçık et al. [9]. Additionally, Bland‐Altman analysis showed good concordance between methods, with 95.6% of paired samples within limits of agreement.

Six‐month TTR was used as a longer measure of INR control. Despite concerns over POCT in APS, no significant difference was observed between 6‐month TTRs of patients self‐monitoring compared to those using venous‐only INR measurements. Mean 6‐month TTRs for both APS cohorts was substantially lower than the non‐APS cohort, supporting previous findings that it is difficult to consistently maintain INR within target ranges in APS [3]. However, it is encouraging that self‐testing produced comparable TTRs to venous‐only testing amongst patients with APS. Review of bleeding complications was not planned as part of the study protocol, but briefly, there was no obvious difference in major bleeding episodes between groups. Minor bleeding was not reviewed.

Currently there lacks a formal systematic evaluation regarding the reliability of POCT for INR monitoring in the setting of APS. Our practise has been to consider use of POCT if there is correlation between five discrete paired POCT and venous INRs with less than ≤0.5 variation in INR values taken within the same 24 h, as well as repeated paired venous sampling every 3–6 months. Our approach has been similar to that highlighted in a recent review by Cohen et al. [11], which additionally details the need for regular and recurrent internal quality control and external quality assessment [8, 12]. The practise of recalling patients with INR readings ≥4.8 is supported by Kalçık et al., who recommended recalling for laboratory measurement at higher INRs due to similarly finding increasing discrepancy between methods as INR increased [9]. Receiving UK‐wide and international referrals for APS as a central London‐based tertiary centre, sampling and thus the results of this study at GSTT is likely representative of the APS population.

POCT empowers patients to be actively involved in their care and is associated with an improved quality of life and therefore may lead to better compliance with warfarin [2]. Self‐monitoring is linked to a higher testing rate compared to routine venepuncture, which facilitates patients to more frequently remain in their therapeutic range [13], thus reducing the risk of complications of mismanaged INRs. Safety‐netting remains essential if the INR falls outside the therapeutic range, in which case venepuncture may offer greater accuracy to modify warfarin dosing as necessary. In patients reporting higher INRs (≥4.8) where disparity between methods may increase, our results support recalling for clinical evaluation.

## 5 CONCLUSION

Our study is among the first to establish the reliability of POCT in this patient group and demonstrates that POCT may be a valid method of monitoring VKA therapy in carefully selected patients with APS, but further study is required in a larger patient cohort. With the rise in telemedicine accelerated by the COVID‐19 pandemic, our results are encouraging and may promote the wider use of POCT in the remote management of APS as a long‐term condition.

## FUNDING INFORMATION

The authors received no specific funding for this work.

## CONFLICT OF INTEREST

The authors declared no potential conflicts of interest with respect to the research, authorship and/or publication of this article.

## AUTHOR CONTRIBUTIONS

All authors made a significant contribution to the manuscript, and this manuscript has not been submitted for publication elsewhere. MM, AL, ES, JM, ZM and KB contributed to the design of the study. MM, AL, ES and JM contributed to the acquisition of data. MM, AL, ES, JM, and ZM contributed to the analysis and interpretation of data. MM, AL and KB contributed to writing and revising the manuscript.

## ETHICS STATEMENT

This manuscript does not contain human studies or experiments using animals. Data was collected retrospectively from routine care and registered as an audit.

## Data Availability

The data that support the findings of this study are available from the corresponding author upon reasonable request.
